# Day-to-Day Contact and Help Among Neighbors Measured in the Natural Environment

**DOI:** 10.1093/geroni/igaa009

**Published:** 2020-04-22

**Authors:** Alexander Seifert

**Affiliations:** 1 Institute of Sociology, University of Zurich, Switzerland; 2 University Research Priority Program (URPP) “Dynamics of Healthy Aging”, University of Zurich, Switzerland

**Keywords:** Contact, Mobile data collection, Neighborhood, Neighboring, Older adults

## Abstract

**Background and Objectives:**

Empirical evidence suggests that the concept of “neighboring” (i.e., social contact and social support within a neighborhood) is related to between-person differences in well-being among older adults. However, little is known about the within-person differences in older adults’ everyday lives, which limits the ecological validity of prior findings. This study examined within-person associations between neighboring and the existence of positive valence, loneliness, and attachment to one’s neighborhood.

**Research Design and Methods:**

The sample consisted of 4,620 observations of 20 days, drawn from 77 adults aged between 61 and 90 years. A mobile application on a smartphone was used for data collection.

**Results:**

The results of the multilevel analysis suggest that daily contact with one’s neighbors was not significantly associated with daily positive valence, but it was positively related to daily feelings of not being alone and daily attachment to one’s neighborhood.

**Discussion and Implications:**

The study makes noteworthy contributions to the field of gerontology by applying a micro-longitudinal research design to assess real-life within-person information.

**Translational Significance**

This study examined how often neighborhood-based contact and help among neighbors occur in the daily lives of older adults. Smartphones were employed to collect daily information about social contact with neighbors. Such contact was positively related to daily feelings of not being alone and daily attachment to one’s neighborhood.

Keller (1968) conceptually divided neighborhoods into the elements of neighbor, neighboring, and neighborhood. The term “neighbor” defines a person’s role (behavior) as a neighbor as well as the attitudes, expectations, and negotiations related to interactions with neighbors, while “neighboring” refers to the social activities in which neighbors engage. The territorial term “neighborhood” describes a spatial area that is physically and symbolically different from the greater environment (Keller, 1968). Neighborhoods are physically bordered spaces in which neighbors live. For gerontological research, neighborhoods are important “places of aging” (Gardner, 2011) because these places contain natural networks of social exchange and support (Kaspar, Oswald, & Hebsaker, 2015). Furthermore, neighbors are defined as social groups whose members interact primarily owing to the commonality of their place of residence. Neighbors can also be evaluated as friends. Therefore, different categories of relationships can fit into the definition of “neighbor,” yet the common ground is locally defined proximity (Hamm, 2000).

This study focused on neighboring as a form of social activity that occurs in a neighborhood. Keller (1968) defined neighboring as “activities engaged in by neighbors as neighbors and the relationships these engender among them” (p. 29). Farrell, Aubry, and Coloumbe (2004) noted that the core component of the concept of neighboring is the consideration of social contact and social support within a given neighborhood. Social contact can range from no contact or short interactions, such as brief greetings or opening the door for neighbors to close contact, such as having long interactions with a high level of trust and reciprocity. Unger and Wandersman (1985) divided social support into personal or emotional support, instrumental support, and informational support. The present study focused on social contact and support among neighbors experienced on a daily basis within an older population.

The exchange of support among neighbors can have an effect on people’s personal social capital, which helps people master their everyday activities (Henning & Lieberg, 1996; Murayama et al., 2015; Redshaw & Ingham, 2017). Hoogerbrugge and Burger (2018) asserted that neighborhood-based social capital is important mainly for those who are the likeliest to spend a considerable amount of time in the neighborhood, especially those who are retired or are in poor health. Thus, older adults—mainly those aged 65 and older and who are retired and/or are in poor health—are an especially interesting group to examine in studying daily contact and help among neighbors. Previous research has shown that help among older adult neighbors is reciprocal, meaning that there is a mutual exchange between help given and help received (Farrell et al., 2004). This reciprocal exchange is driven by personal characteristics, health resources, living situation, social factors, and contextual factors (Seifert & König, 2019).

According to Cantor (1979), the domestic environment becomes more important in old age, primarily because of personal limitations, such as health, mobility, and social networks, and also place attachment (Lawton & Nahemow, 1973; Wahl, Iwarsson, & Oswald, 2012). Older adults spend a great deal of time in their homes as well as in their neighborhoods (York Cornwell & Cagney, 2017). Shaw (2005) demonstrated that the expectation of support from neighbors is strongest among older adults. This is primarily because they have more frequent contact with their neighbors and more residential stability than younger adults (Glass & Balfour, 2003; Heinze, Kruger, Reischl, Cupal, & Zimmerman, 2015). This expectation of support becomes even more crucial in times of declining fertility rates, smaller family sizes, and greater mobility (Brandt, Haberkern, & Szydlik, 2009; Isengard & Szydlik, 2012). While help from family members might be limited, help from friends or neighbors is more accessible and, therefore, maybe more important (Boerner, Jopp, Park, & Rott, 2016; Deindl & Brandt, 2017; Greenfield & Reyes, 2015). Having ties to neighbors facilitates access to informal aid and reduces people’s sense of isolation. Oswald, Jopp, Rott, and Wahl (2011) noted that such ties could mitigate problems of maintaining everyday life at a time of advancing age and declining health. Overall, the positive effects of social contact on the well-being of older adults are well documented (Farrell et al., 2004; Schwirian & Schwirian, 1993; Wenger, 1990).

However, does daily contact with neighbors positively influence the well-being of older adults? Social needs theories, such as the social convoy model (Kahn & Antonucci, 1980), claim that social network size decreases with age, but older adults maintain their existing interactions and use network partners that are easy to reach. For example, when physical limitations restrict a person’s mobility, neighbors gain more importance because of their spatial proximity. Furthermore, socioemotional selectivity theory (Carstensen, 1993) indicates that older adults seek out interactions that positively influence their well-being. This selection depends on their degree of emotional closeness and the emotional gratification that these interactions provide. Older adults can decide when and from whom to accept help, and this may strengthen their sense of control and self-competence. Besides immediate relatives and friends, peripheral relationships, for example, with neighbors, can be evaluated as emotionally close. Contact with neighbors is, therefore, also selective in the sense that relationships with neighbors will be maintained if they have a positive effect on well-being. Furthermore, this means that such selective relationships can directly affect daily well-being.

Previous studies, such as those of Boerner and colleagues (2016), Kalwij, Pasini, and Wu (2014), and Wang, Chen, Shen, and Morrow-Howell (2018), have shown that contact with neighbors can mitigate the problems of coping with everyday life at a time of advancing age and declining health. Reciprocal social contact and support can protect individuals from negative life stress (Ingersoll-Dayton, Morgan, & Antonucci, 1997) and positively affect their mood (Greenfield & Reyes, 2015; Williamson & O’Hara, 2017). This contact with our neighbors can affect whether we feel well and content, which is known in psychology as the mood dimension of positive valence (Wilhelm & Schoebi, 2007). No known studies to date have investigated this relationship between contact with neighbors and positive valence in the day-to-day context of older adults.

Similar results may be observed regarding the feeling of not being lonely, which is also an important outcome of social contact, as Holt-Lunstad, Smith, Baker, Harris, and Stephenson (2015) pointed out. Previous studies, such as those of Kearns, Whitley, Tannahill, and Ellaway (2015) and Pinquart and Sörensen (2001), have generally found positive associations between having social contact with neighbors and feeling less alone in old age, although no known studies have investigated this relationship using a microlongitudinal approach among older adults. Therefore, data relating to the day-to-day assessment of the relationship between loneliness and contact with neighbors are missing.

In addition to determining the outcomes of positive valence and the feeling of not being alone, it would also be worthwhile to look at a more environment-related relationship between contact with one’s neighbors and attachment to one’s neighborhood. Within the field of gerontology, Wahl and colleagues (2012) have spoken of belonging in their evaluation of place attachment among older adults. The authors showed that the processes of housing-related belonging were connected both to autonomy and well-being (Bruggencate, Luijkx, & Sturm, 2018; Cramm & Nieboer, 2015; Oswald et al., 2007; Zhang, Zhang, Zhou, & Yu, 2018). While those previous studies showed that a feeling of belonging to a neighborhood contributes to well-being in old age, no known studies have investigated the within-person effect of daily contact on one’s attachment to a neighborhood. The present study examined whether such contact might exert a positive influence on people’s personal attachment to their neighborhood.

Thus, numerous authors have discussed older adults’ neighboring and the relationship between neighboring and well-being. However, the findings are limited to information aggregated across a whole range of real-life situations; in real life, the functional effect of neighboring depends on the situation and contextual specifics that should be considered. Another limitation of previous studies, which predominantly used cross-sectional interviewing for data collection, is that when people were asked to reflect on what they typically did (such as how many neighbors they typically met), they were likely to draw on lay theories or recent memories or venture guesses. Therefore, measuring neighboring should involve collecting information from real-life contexts and situations. Experience sampling and daily diary methods can be used to repeatedly assess people’s daily activities and their situational contexts across a wide range of real-life contexts (Brose & Ebner-Priemer, 2015; Seifert, Hofer, & Allemand, 2018). Another advantage of such methods is that their findings will be ecologically valid because they were collected during people’s daily lives and, thus, capture behaviors and experiences in real environments outside of research laboratories. Intensive repeated measurements of one participant can be used to capture within-person information, which will allow for studying the situational contexts, mechanisms, and processes that underlie behavior, and such information may contrast with between-person information (Hamaker, 2012).

Presumably, no studies to date have investigated daily contact and the offer of help exchange with neighbors among older adults using an experience sampling approach over a period of time. Therefore, there is a need for a new methodological approach involving a strong consideration of concrete day-to-day behaviors in natural environments (Wahl & Gerstorf, 2018).

## The Present Study

According to the ecological model of aging (Lawton & Nahemow, 1973; Wahl and colleagues, 2012), one’s social and physical environments, such as those found in a neighborhood, have the potential to enhance opportunities for aging well, as contact with neighbors supports the management of daily life and feelings of not being alone and attachment and overall well-being. Previous evidence showed that the exchange of support among neighbors positively affects one’s personal social capital, which helps older adults master their everyday activities (Henning & Lieberg, 1996; Hoogerbrugge & Burger, 2018; Murayama et al., 2015; Redshaw & Ingham, 2017). Therefore, the present study investigated whether the theoretical and empirically based positive effects of neighboring could be found in the daily life of older adults. Accordingly, this study examined within-person associations of 20 days between neighboring and the existence of positive valence, loneliness, and attachment to one’s neighborhood. The research questions were as follows:

1. How often are contact with neighbors and help exchange among neighbors reported in daily life?
2. What are the between-person predictors of the frequency of occurrence of contact and help among neighbors?
3. What are the within-person associations of contact with one’s neighbors and positive valence, loneliness, and attachment to one’s neighborhood?

This intensive, microlongitudinal study focused on daily contact and help exchange among neighbors in an older population during a 20-day period. I used an experience sampling method (Brose & Ebner-Priemer, 2015; Harari et al., 2016; Seifert et al., 2018) and conducted assessments three times daily using a smartphone with an application for self-report questionnaires to study the everyday lives of older adults. First, I was interested in the frequency of daily contact and daily help that occurs among neighbors as well as the between-person factors of the breadth of social interaction in neighborhoods (i.e., contact and help). Second, I focused on within-person associations between contact with neighbors and positive valence, loneliness, and attachment to one’s neighborhood. I expected neighborhood contact and positive valence to show day-to-day variations within individuals and to be positively interrelated. Furthermore, I expected participants to report more positive valence during events in which they have more contact with neighbors than usual.

In addition to examining the relationships between neighbor contacts and positive valence, I explored the relationship between neighbor contacts and loneliness and expected them to be negatively interrelated. Moreover, I expected participants to report less loneliness during events when they have greater contact with neighbors than usual. I was also interested in examining the relationship between neighborhood contact and attachment to one’s neighborhood, and I expected to observe a positive interrelationship between them. I anticipated that participants would report more attachment to their neighborhood during events when they have more contact with their neighbors than usual.

## Research Design and Methods

### Participants

A total of 77 healthy older adults aged between 61 and 90 years (*M* = 72.45; standard deviation [*SD*] = 7.11; 62.3% women; 98.7% with Swiss citizenship) participated in this study. All participants lived in private households (48.1% lived alone; 74.2% lived in a rented apartment/house). Of the total, 20.8% of participants were single, 48.0% were married or in a relationship, 18.2% were divorced or separated, and 13.0% were widowed. Most participants (62.3%) had children, of which 16.3% lived in the same neighborhood, 40.8% lived in the same city, and 42.9% lived elsewhere. Most participants (93.5%) were retirees or early retirees. Furthermore, 40.3% of participants had tertiary education, and 32.4% had a monthly household income of less than 4,500 Swiss francs (CHF; equivalent to roughly USD 4,565 or EUR 3,990), whereas 16.9% had an income of more than CHF 10,500.

Participants reported overall good subjective health of 5.05 (*SD* = 0.77), measured on a six-point Likert scale (1 = *very bad* to 6 = *very good*). They also reported good overall autonomy, with an average value of 3.51 (*SD* = 0.51) on the four-item, four-point Perceived Autonomy in Old Age scale (Cronbach’s *α* = .74), which operationalizes participants’ subjective evaluation of their independence and freedom of choice (Schwarzer, 2008).

### Procedure

The study took place from July to November 2017 among participants from the city of Zurich, Switzerland. Participants were recruited from the participants’ pool of the University Research Priority Program, “Dynamics of Healthy Aging,” through announcements in local newspaper articles and flyers posted at public social facilities. The requirements for participating in the study were that older adults had to (a) be mobile, (b) be cognitively able to pass (>25 points) the Mini-Mental State Examination (Folstein, Folstein, & McHugh, 1975), and (c) not live in a nursing home or require daily care. A further requirement was that participants (d) be had to have either used or had experience with a mobile phone. The study was approved by the ethics committee of the Faculty of Philosophy of the University of Zurich (approval number 16.8.4).

Initially, participants filled out an informed consent form and a baseline questionnaire with demographic variables and scales concerning their neighborhood situations and personal characteristics. In the first meeting, the purpose and procedure of this study and smartphone usage were explained, and participants had the opportunity to ask questions. An in-depth, four-level smartphone training protocol (verbal overview, demonstration, participant testing, and practical handout) was developed and administered to the participants to maximize data quality and adherence. Furthermore, a support hotline (operated by the author) was provided for reporting any technical problems or asking questions.

The intensive, longitudinal part of the study commenced after the first meeting. During this period, participants carried a loaned Android smartphone (Motorola Moto E, second generation) equipped with the “movisensXS” (Version 1.1.1; movisens GmbH, Karlsruhe, Germany) ecological momentary assessment application. The application was designed to sound an alarm three times a day, randomized in the following time frames: 9 a.m. to 12 p.m., 1 p.m. to 4 p.m., and 5 p.m. to 8 p.m. On hearing the alarm, participants were required to answer a series of questions on the smartphone about social contact (especially with their neighbors) during the previous 3 hr as well as their current mood and attachment to their neighborhood.

At the end of the study, the participants returned the smartphones and were asked about any problems that they may have encountered with data entry. The meeting days were not included in the data analyses. The study yielded data from 20 days and 77 participants, who provided a total of 4,620 measurement points. Participants missed entering data on 455 alarms, which yielded a 9.8% missing data rate (only three participants had more than 30% missing data). At the end of the study, participants received an expense compensation of CHF 100 and a written report of their overall contact within the neighborhood and mood profiles during the study days.

### Dependent and Independent Variables

#### Neighbor contact and help

With every new alarm (three times a day), the participants were asked which of the following people they had contacted in the previous 3 hr, either alone or with another person: their partner, children, grandchildren, other relatives, friends, and neighbors. When they answered “neighbors,” they were asked whether this interaction had taken more than 2 min. Whenever this question was answered positively, it was categorized as “intensive contact” (i.e., more than just saying hello on the stairs), which will be referred to as “contact” (yes/no) in the following. After answering the contact questions, participants who had contacted their neighbors also answered a question about whether they had helped a neighbor (yes/no) or if they had received help from a neighbor (yes/no) in the past 3 hr.

#### Positive valence, loneliness, and attachment to one’s neighborhood

Positive valence was taken from Wilhelm and Schoebi’s (2007) Short-Scale to Measure Three Basic Dimensions of Mood. The study focused on the “valence” dimension. During each observation, participants responded to the statement, “At this moment, I feel …,” by means of two bipolar items, which were presented in the following order in one display: “content–discontented” and “unwell–well.” The scales comprised five points (the endpoints were associated with the label “very”). Prior to the analyses, the data from the first item were reverse-coded to ensure that higher scores would indicate higher positive valence. The calculated scale variable of positive valence (*M* = 4.33; *SD* = 0.74) had a Cronbach’s α of .75.

Loneliness was measured in the same way as positive valence [i.e., using Wilhelm and Schoebi’s (2007) Short-Scale to Measure Basic Dimensions of Mood] using a bipolar item on a five-point scale: “At this moment I feel … alone–not alone.” The use of data from this item (*M* = 4.02; *SD* = 1.26) ensured that higher scores would indicate higher values of feeling not alone.

The notion of attachment to one’s neighborhood was inspired by Lalli’s (1992) work and was measured in the same way as positive valence and loneliness. A bipolar item with a five-point scale was used: “At this moment I feel … attached to my neighborhood–not attached to my neighborhood.” Data from this item (*M* = 2.38; *SD* = 1.36) were reverse-coded to ensure that higher scores would indicate higher levels of attachment to one’s neighborhood.

#### Control variables

The following factors for each participant were included to control for between-person differences from the baseline questionnaire: (a) age (in years), sex (0 = *male*; 1 = *female*), household income (scale), living alone (0 = *no*; 1 = *yes*), childlessness (0 = *no*; 1 = *yes*), and duration of residence in the neighborhood (in years); (b) scores from the WHO-5 Well-Being Index (Topp, Østergaard, Søndergaard, & Bech, 2015); (c) health status, using the four items of “mobility,” “self-care,” “usual activities,” and “pain/discomfort” from EuroQol Group’s (1990) EQ-5D scale; (d) importance of social contact (“Social contact is important for me”), measured on a four-point Likert scale; (e) people’s prioritization of neighbor contact (“What value do your neighbors have in your social network?”), measured on a scale from 1 = *no value at all* to 6 = *very high value*; and (f) overall “neighboring” in the neighborhood, through the question “How would you describe the neighborhood relationship in your neighborhood?” measured on a scale from 1 = *Nobody knows each other in my neighborhood* to 4 = *The neighbors in my neighborhood are really good friends*. Table 1 shows the ranges, average values, and SDs of all control variables.

**Table 1.** Multiple Regressions to Predict Total Count Variables of Contact and Help

	Descriptive	Model 1: Contact with neighbors (counta)		Model 2a: Help given (count)	Model 2b: Help received (count)
	Min–max, mean (*SD*)	1 Predictor models (*β*, *t*) [*R*^2^]	Full model (*β*, *t*)	Full model (RR)	Full model (RR)
Age (years)	61–90, 72.45 (7.10)	.23, 2.06* [.05]	.23, 1.62	1.04	1.08*
Sex (female)	0–1, 0.62 (0.48)	.10, 0.90 [.01]	.12, 1.05	3.07**	1.36
Household income (scale)	1–7, 3.83 (1.78)	−.04, −0.30 [.00]	−.06, −0.45	1.24	1.23
Living alone (yes)	0–1, 0.48 (0.50)	−.01, −0.08 [.00]	−.07, −0.46	1.96	1.21
Childlessness (yes)	0–1, 0.37 (0.48)	−.23, −2.13* [.05]	−.05, −0.35	0.37	0.80
Duration of residence in neighborhood (years)	1–82, 26.68 (18.20)	.11, 0.94 [.01]	.02, 0.13	0.98	0.97
WHO-5 (scale)	5–25, 18.63 (3.91)	.09, 0.80 [.01]	.16, 1.37	1.10	1.09
Health status (scale)	3–12, 10.98 (1.40)	−.05, −0.48 [.00]	−.03, −0.26	0.95	1.07
Importance of social contact (scale)	1–4, 3.26 (0.94)	0.14, 1.26 [.02]	.12, 0.97	1.06	0.84
Prioritization of neighbor contact (scale)	1–6, 4.13 (1.12)	0.44, 4.30*** [.20]	.30, 2.31**	1.53*	1.20
Overall “neighboring” in neighborhood (scale)	1–4, 2.80 (0.58)	0.28, 2.59* [.07]	.17, 1.42	0.99	1.66
*Model accuracy*			*N* = 75 *R*^2^ = .32	*N* = 75 AIC = 206.45 BIC = 233.60	*N* = 75 AIC = 167.27 BIC = 194.43

*Note:* AIC = Akaike information criterion; BIC = Bayesian information criterion; missing values included listwise; *β* = standardized coefficient; *t* = *t* value.

^a^Square root of aggregated contacts counts.

*p < .05. **p < .01. ***p < .001.

Additionally, for contextualization the social contacts of the participants within the descriptive results, the questionnaire included the following variables: (a) daily contact with another person (three times a day, the participants were asked which of the following people they had contacted in the previous 3 hr: their partner, children, grandchildren, other relatives, friends, or no one; resulting in each case in a 1 = *contact* and 0 = *no contact* dummy variable); (b) evaluation of neighbor contact (measured on a 5-point Likert scale ranging from 1 = *very unpleasant* to 5 = *very pleasant*; (c) place of meeting with neighbors (when contact with neighbors had taken place, participants were asked where they had met their neighbors (e.g., shared laundry room, neighbor’s apartment, participant’s apartment, in the hallway, within the participant’s residential building, within the participant’s neighborhood; outside the participant’s neighborhood; resulting in each case in a 1 = *yes* and 0 = *no* dummy variable); (d) circumstances of meeting with neighbors (randomly, participants had sought contact with their neighbors, neighbors had sought contact with the participant; resulting in each case in a 1 = *yes* and 0 = *no* dummy variable); (e) reasons for meeting with neighbors (asking for help, asking about current condition, simply “a nice chat,” no particular reason; resulting in each case in a 1 = *yes* and 0 = *no* dummy variable); and (f) evaluation of received help from neighbors (measured on a five-point Likert scale ranging from 1 = *not helpful at all* to 5 = *very helpful*.

### Statistical Analysis

First, explorative findings regarding contact and help among neighbors were derived through descriptive statistics. Second, a linear regression was done using the count variable (more precisely, the square root of aggregated contacts for each participant) of “contact with one’s neighbors” as the dependent variable to predict if sociodemographic variables (age, sex, income) and personal situation (living alone, health, well-being) influence the frequency of contact with one’s neighbors (Table 1). Third, two log-linear models for count data (using negative binominal regression because of over-dispersed count data) were calculated to analyze the between-person predictors of help given and received among neighbors (Table 1). Fourth, Pearson correlations were used to examine the relationships among the main study variables (Table 2). Finally, within-person associations between contact with one’s neighbors and positive valence, loneliness, and attachment to one’s neighborhood were investigated. In contrast to most conventional quantitative longitudinal models, the intensive real-time study design provided multiple measurements of self-reported observations to capture within-person processes. As multilevel modeling allows for analyzing such a data structure in which multiple measurements are nested within a person, multilevel analyses (Bolger & Laurenceau, 2013) were used to investigate the within-person-related research questions. For the analytical procedure, the nested structure of the data was examined by computing intraclass correlation coefficients (ICC) and comparing the extent of the total variance of a within-person variable (Table 2). Subsequently, the associations between daily contact with one’s neighbors and positive valence (Table 3) were analyzed while controlling for the person-mean of contact, age, sex, contact with others (partner, children, grandchildren, other relatives, friends), and time (Model 1). The same procedure was applied for loneliness (Model 2) and attachment to one’s neighborhood (Model 3; Table 3). SPSS version 24 (IBM Statistics, Armonk, NY) was used for the statistical analysis.

**Table 2.** Pearson’s Correlations and Estimates of Null Models

	Range	*M*	*SD*	1	2	3	ICC	% of total variance within-person
1. Contact with neighbors	0–1	0.16	0.36				.10	90.03
2. Positive valence	1–5	4.32	0.73	.05**			.48	51.59
3. Feeling not alone	1–5	4.02	1.26	.04*	.27***		.45	54.91
4. Attachment to one’s neighborhood	1–5	2.38	1.36	.25**	−.02	.08***	.46	54.35

Notes: *N* = 4,099–4,620. ICC = intraclass correlation coefficient; *M* = mean, *SD* = standard deviation.

*p < .05. **p < .01. ***p < .001.

**Table 3.** Multilevel Analyses With Fixed and Random Effects for Contact With Neighbors

	Model 1: Positive valence		Model 2: Feeling not alone		Model 3: Attachment to one’s neighborhood	
	1 Predictor model estimate (*SE*)	Full model estimate (*SE*)	1 Predictor model estimate (*SE*)	Full model estimate (*SE*)	1 Predictor model estimate (*SE*)	Full model estimate (*SE*)
Fixed effects						
Intercept	4.27*** (.06)	4.10*** (.10)	3.95*** (.10)	3.45*** (.15)	2.47*** (.11)	2.05*** (.16)
Contact with neighbors	.05 (.03)	.05 (.03)	.13** (.43)	.11* (.44)	.70*** (.07)	.68*** (.07)
Person-mean contact with neighbors		.63 (.49)		.35 (.74)		2.70** (.78)
Contact with others (partner, children, grandchildren, other relatives, friends)		.11 (.02)***		.63*** (.04)		−.01 (.04)
Age		−.02 (.01)		−.04** (.01)		.01 (.01)
Sex (female)		<.01 (.12)		.17 (.18)		.34 (.19)
Time	<.01 (<.01)	<.01 (<.01)	<.01 (<.01)	<.01 (<.01)	<.01 (<.01)	<.01 (<.01)
Random effects						
Intercept	.26***	.25*** (.04)	.72*** (.12)	.58*** (.11)	.08*** (.13)	.65 *** (.11)
Contact	.01 (.01)	.01 (.01)	<01 (.01)	.01 (.01)	.23*** (.06)	.22** (.06)
Residual	.28*** (.01)	.28*** (.01)	.89*** (.02)	.83*** (.02)	.93*** (.02)	.93*** (.02)
AIC / BIC	6,731.64 / 6,763.31	6,716.47 / 6,748.14	11,417.60 / 11,449.20	11,147.07 / 11,178.66	11,510.03 / 11,541.62	11,504.07 / 11,535.66

*Notes: N* = 77 people, three times a day, 20 days; 4,620 total observations. Age and sex (*male* = 0; *female* = 1) are grand mean-centered. Time reflects the ordinal time point (measurement points, 0–59); AIC = Akaike information criterion; BIC = Bayesian information criterion; *SE* = standard error.

*p < .05. **p < .01. ***p < .001.

## Results

### Descriptive Findings Regarding Contact and Help Among Neighbors

When all valid measurement points from the 77 participants over 20 days were considered, the participants had seen a neighbor in 790 cases (18.9%). In comparison, in 32.2% of all cases, participants had contact with friends. In 76.3% of all cases, participants who lived with a partner reported having had contact with that partner. In 12.6% of all cases, participants who had children reported having had contact with their children. In 17.0% of all cases, participants reported having been alone during the preceding 3 hr.

After conducting a general assessment of the contact, the participants were asked if this contact with a neighbor had lasted for more than 2 min, which would indicate contact beyond simply saying hello. Such contact was reported in 658 (15.7%) valid cases. In the following, the term “contact with one’s neighbor” is used to denote these events. The meeting had a duration of less than 11 min in 44.2% of all cases, from between 11 and 60 min in 50.9% of cases, and for more than 60 min in 4.9% of cases. The contact occurred more often in the evening (17.2%) than in the morning (13.8%). Overall, the participants evaluated their contact with neighbors positively: they reported an average score of 4.55 (*SD* = 0.63); note that controlling for skewness in the response choices did not change this result. The calculated sum of all contact with neighbors across the 60 measurement points ranged from 0 to 32, with an average of 8.54 (*SD* = 6.78).

When contact with neighbors had taken place, the participants were asked (with multiple replies possible) where they had met their neighbors. Sorted by frequencies, the contact took place (a) in a shared laundry room (4.6%); (b) at a neighbor’s apartment/residence (13.2%); (c) at the participant’s apartment/residence (16.1%); (d) outside the participant’s neighborhood (20.9%); (e) in the hallway of the participant’s residential building (28.1%); (f) within the participant’s neighborhood (28.2%); and (g) in the outdoor area surrounding the residential complex (29.8%).

In 54.6% of cases, participants had randomly met their neighbors (i.e., by bumping into each other); in 36.7% of cases, they had sought contact with their neighbors; and in 29.8% of cases, the neighbors had sought contact with them. When no random contact with neighbors had taken place, participants were asked (again with multiple replies possible) what the reason for the meeting had been. Sorted by frequencies, participants reported (a) no direct reason for the contact (14.9%); (b) the neighbor had helped the participant (18.7%); (c) the neighbor had asked about the participant’s current condition (25.6%); (d) the participant had helped the neighbor (30.1%); (e) the participant had asked about the neighbor’s current condition (32.2%); and (f) the participant reported simply having had “a nice chat” (38.1%).

The calculated sum of giving help to one’s neighbors across the 60 measurement points ranged from 0 to 8, with an average of 1.12 (*SD* = 1.76). The calculated sum of receiving help from one’s neighbors ranged from 0 to 6, with an average of 0.70 (*SD* = 1.23). Overall, the participants evaluated receiving help from their neighbors positively: on a five-point scale from 1 = *not helpful at all* to 5 = *very helpful*, they reported an average of 4.30 (*SD* = 0.97).

### Multivariate Findings for Contact and Help With Aggregated Observations

Table 1 displays the linear regression results on the left-hand side (Model 1), with the count value of “contact with one’s neighbors” as the dependent variable. Age, sex, household income, living alone, childlessness, duration of residence, the WHO-5 Well-Being Index, health status, importance of social contact, people’s prioritization of contact with neighbors, and overall “neighboring” in the neighborhood were included. The test of the full model was statistically significant [*F*(11, 64) = 2.490; *p* = .012]. The full model showed that only people’s prioritization of contact with neighbors significantly contributed to the prediction, whereas the other independent variables were not predictors in the multivariate analysis. The participants who prioritized contact with neighbors, in general, were predominantly those who had more frequent contact with their neighbors.

The right-hand side of Table 1 provides the results of two negative binomial regressions. In Model 2a, the count of “help given” was considered the dependent variable. The same independent variables as in Model 1 were included. The test of the full model was statistically significant [χ ^2^ (11; *n* = 75) = 27.60; *p* = .004]. The full model showed that (a) being woman and (b) prioritization of contact with neighbors both significantly contributed to the prediction, whereas the other independent variables were not significant predictors. Women and participants who prioritized contact with neighbors, in general, tended to be those who provided frequent help to their neighbors.

In Model 2b, the count of “help received” was considered the dependent variable, and the same independent variables as before were included. The test of the full model was statistically significant [χ ^2^ (11; *n* = 75) = 17.21; *p* = .043]. The model showed that only age contributed to the prediction significantly, indicating that older participants received help from their neighbors more often than did younger participants.

### Multilevel Findings Among Within-Person Observations

Table 2 displays within-person descriptive statistics and correlations among the variables of interest. The results show that contact with one’s neighbors was positively correlated with positive valence, lack of loneliness (i.e., not feeling alone), and attachment to one’s neighborhood. Positive valence was intercorrelated with loneliness (but not with attachment to one’s neighborhood), and loneliness was intercorrelated with attachment to one’s neighborhood. ICCs were computed to obtain a value that would describe the amount of within-person variance with respect to the total variance. The results show that for contact with one’s neighbors, 90% of the total variance was within-person, and 10% was between-person. For positive valence, 52% of the variance was within-person; for loneliness, the variance was 55%; and for attachment to one’s neighborhood, the variance was 54%. Thus, I decided to analyze the data using multilevel modeling.

Model 1 (Table 3) was analyzed, which consisted of the outcome variable “positive valence” and the focal within-person predictor, “contact with one’s neighbors”; the control variables were the person-means of “contact with one’s neighbors,” age, sex, contact with others, and time. According to the results, contact with one’s neighbors was not significantly associated with positive valence from the within-person perspective. The within-person variable of “quality of contact with one’s neighbor” (pleasant–unpleasant) and the person-mean variable of “quality of contact” were additionally added to Model 1b and a significant association between the quality of contact (*b* = .16; *p* = .016) and positive valence was found. Therefore, contact alone does not predict positive valence, but the additional quality of the contact does.

Then Model 2 (Table 3) was analyzed, which consisted of the outcome variable, “lack of loneliness (not feeling alone)”; the focal predictor was “within-person contact with one’s neighbors”; and the control variables were person-means of contact with one’s neighbors, age, sex, contact with others, and time. The findings show that contact with one’s neighbors was positively associated with within-person “not feeling alone,” meaning that contact with neighbors was associated with a decrease in loneliness.

Finally, in Model 3 (Table 3), the outcome variable “attachment to one’s neighborhood” was tested with respect to the predictor, “contact with one’s neighbors,” under the control of the person-means of “contact with one’s neighbors,” age, sex, contact with others, and time. The results show that contact with one’s neighbors was positively associated with attachment to one’s neighborhood, meaning that contact with one’s neighbors was associated with an increase in feeling attached to one’s neighborhood.

Moreover, six additional models with the outcome variables, “positive valence,” “loneliness,” and “attachment to one’s neighborhood,” and the predictors, “help given to neighbors” and “help received from neighbors,” were tested. Only the two models with the outcome variable “attachment to one’s neighborhood” showed a significant and positive association between “help given” (*b* = .60; *p* = .005) and “help received” (*b* = .61; *p* = .009) and “attachment to one’s neighborhood,” meaning that participants who give or receive help feel more attached to their neighborhood.

## Discussion

This microlongitudinal study was one of the first to examine contact with neighbors within 20 days and to look at the within-person associations between contact with one’s neighbors and positive valence, loneliness, and attachment to one’s neighborhood in the everyday lives of healthy older adults.

First, the descriptive findings show that the participants had relatively rare contact with their neighbors in comparison to contact with friends, although the results also show that this contact with neighbors occurred more often than contact with their own children. When contact with neighbors occurred, such contact often lasted for more than 10 min, meaning that these interactions were few in number but intensive. Furthermore, the participants evaluated their contact with neighbors positively. For these reasons, contact with one’s neighbors should not be neglected, because such contact is important for social interaction in the everyday lives of older adults. Previous studies have indicated that such neighborhood interaction can positively affect older adults’ ability to cope with daily activities (Cain, Wallace, & Ponce, 2018; Cramm, van Dijk, & Nieboer, 2013). Even if neighbors do not represent strong ties, it is sometimes easier to spontaneously accept or provide support without regard for any repercussions or complications (Aral, 2016).

The results of the present study also show that when participants either gave or received help from their neighbors, they provided more help than they received. Thus, older adults should not be viewed only as recipients of help in caring communities but also as providers of help (Hand, Laliberte Rudman, Huot, Pack, & Gilliland, 2018). Local social projects would likely improve neighborhood assistance in a community by addressing older people to convince them to participate in volunteer work (Scharlach & Lehning, 2013).

Second, the multivariate findings on predictors of the frequency of occurrence of contact and help among neighbors reveal that from the between-person perspective, only the participants’ prioritization of contact with neighbors was a significant predictor for contact with their neighbors. For help given, the analysis showed that women and those who prioritized contact with neighbors provided help the most often, whereas only age was a significant predictor of help received. To summarize this second point, those who had an overall positive attitude toward their neighbors, as measured by their prioritization of contact with neighbors, had more contact and provided more help. Those attitudes toward neighboring, which Keller (1968) referred to as the “neighbor dimension,” reflect the individuals’ valuation of their contact with neighbors, particularly around whether the neighbors are important for their social (and support) network. To ensure contact with neighbors, people must have a positive attitude toward neighboring to initiate such contact or to accept offerings of future contact.

The between-person findings concerning help and women are in line with Brandt and colleagues (2009), in that women are more likely to provide help, especially in terms of emotional support. However, the findings on age for men and woman are also in line with Seifert and König (2019), in that the older study participants received more help from their neighbors than they provided, while the younger participants provided more help than they received. These results corroborate previous findings that one’s neighborhood and help from one’s neighbors both become more important in old age, primarily because of personal limitations (such as health, mobility, and social networks) and place attachment (Kaspar et al., 2015; Shaw, 2005). Nevertheless, it must be emphasized that because the present study included healthy older adults, generalizations about all older adults are limited (as discussed in the Limitation section), and future research should include other groups of older adults.

Furthermore, the daily assessment facilitated the understanding that contact with neighbors varies strongly within older people with equal levels of health and autonomy. It can be also assumed that contact with one’s neighbors, in addition to the between-person finding of positive attitudes toward one’s neighbors, is strongly dependent on potential opportunities (such as chance encounters with neighbors on the stairs or outside the building), or the need for help.

Finally, this was the first study to test within-person associations of contact with one’s neighbors and positive valence, loneliness, and attachment to one’s neighborhood. No direct significant association between contact and positive valence was found, but a moderate association between quality of contact and positive valence was identified. That is, when participants evaluated their contact with neighbors as pleasant, their positive valence was higher. Positive valence had a high intercept value, which means that additional variance explained from contact with one’s neighbors was difficult to find. Furthermore, positive valence was relatively stable (and high). This is in line with the “paradox of subjective well-being” phenomenon where older adults who are functionally limited still feel content (Staudinger, 2000).

Nevertheless, the results show a positive association between contact with neighbors and the feeling of not being alone. In other words, when contact with one’s neighbors had occurred during the preceding 3 hr, the participants felt less alone. This finding is in line with previous research on social contact and loneliness in general (Pinquart & Sörensen, 2001; Queen, Stawski, Ryan, & Smith, 2014). This subjective feeling of not being alone can also have a positive effect on the general well-being of older adults and can decrease the feeling of social exclusion within a neighborhood (Dahlberg & McKee, 2018; Finlay & Kobayashi, 2018).

A positive association between contact with one’s neighbors and attachment to one’s neighborhood was also found in this study. When controlling for within-person, the findings show that the feeling of connection (attachment to one’s neighborhood) increases during the period when contact with neighbors occurred. Known as “place identity” (Proshansky, Fabian, & Kaminoff, 1983), the feeling of attachment to one’s neighborhood gave participants a self-identity that consisted of cognition about the physical world in which they live. Wahl and colleagues (2012) described this within the field of gerontology as belonging to a neighborhood—a belonging that Morita, Takano, Nakamura, Kizuki, and Seino (2010) indicated relates both to autonomy and well-being. The results show that contact with one’s neighbors in everyday life has a positive within-person effect on one’s feeling of belonging to a neighborhood. Such contact, therefore, can affect one’s attachment to the neighborhood, and the converse may also be true: attachment to a neighborhood is important for ensuring active interaction within the social component of that neighborhood.

### Limitations

Despite its strengths, this study had several limitations. First, the study focused on 77 participants for 20 consecutive days. Although I am confident that the study provided an idea of the associations between the examined processes, I cannot deny that the use of more participants (and also more people with low health status) and days would be necessary to obtain a more valid picture of the everyday lives of older adults in a neighborhood. Future researchers should assess a larger number of participants over a longer assessment period to examine more between-situation aspects. Second, both time- and event-triggered assessments should be applied; in other words, participants would report on their interactions with neighbors once a certain interaction has occurred. Third, only two items to capture positive valence and one item each for loneliness and attachment to one’s neighborhood were used in the study. Future researchers may want to use more items to capture the underlying theoretical constructs in greater detail. Fourth, because the study has not provided data on important social network variables, such as emotional closeness or contact biographies, researchers should include such variables in an ambulatory assessment approach. Fifth, this study collected no information about the ages of neighbors who provided help to participants; therefore, the results do not reveal anything about the age-related exchange of help or contact. Sixth, this study did not look at which kinds of neighbor interactions were most helpful and meaningful in different contexts; future research should include more contextual information, such as stress or happiness level of participants and weather or the actual political situation in the area where participants live. Seventh, this study focused on relationships between contact with neighbors and positive valence, loneliness, and attachment to the neighborhood; however, future research should also consider the opposite pathway, where individuals who are lonely do not seek contact with their neighbors. Eighth, differences between different social ties (e.g., friends) and frequency of contact with neighbors within and between days were not investigated in this study; this should be addressed in future research. Finally, multilevel models were used to look in only one direction—from contact with one’s neighbors toward positive valence, loneliness, and attachment to one’s neighborhood—but lower valence or less loneliness could potentially lead to greater contact with one’s neighbors. Future studies could disentangle these bidirectional relationships.

### Implications

Partly pronounced contact with neighbors and help exchanges exist among older adults, even if such contact is less frequent than that with family or friends. The between-person perspective of this study has yielded results demonstrating that a positive attitude toward neighboring is important for contact with one’s neighbors and for the help people give to their neighbors. The results also support positive within-person associations between contact with one’s neighbors and not feeling alone and feeling an attachment to one’s neighborhood. Future studies should aim to better understand how, when, and why contact with neighbors occurs and which effects such contact has on coping with daily activities and stabilizing the daily well-being of older adults. Overall, this study has added new evidence to the within-person research on neighboring in the everyday lives of older adults.

## Funding

This research did not receive any specific grant from funding agencies in the public, commercial, or not-for-profit sectors.

## Conflict of Interest

None reported.
